# Expert Opinion on the Perceived Effectiveness and Importance of On-Farm Biosecurity Measures for Cattle and Swine Farms in Switzerland

**DOI:** 10.1371/journal.pone.0144533

**Published:** 2015-12-10

**Authors:** Karin Kuster, Marie-Eve Cousin, Thomas Jemmi, Gertraud Schüpbach-Regula, Ioannis Magouras

**Affiliations:** 1 Veterinary Public Health Institute, Vetsuisse Faculty, University of Bern, Schwarzenburgstrasse 155, 3097 Liebefeld, Switzerland; 2 Consumer Behavior, Institute for Environmental Decisions (IED), ETH Zurich, Universitaetstrasse 22, 8092 Zurich, Switzerland; 3 Federal Food Safety and Veterinary Office (FSVO), Schwarzenburgstrasse 155, 3003 Bern, Switzerland; The University of Melbourne, AUSTRALIA

## Abstract

Biosecurity is crucial for safeguarding livestock from infectious diseases. Despite the plethora of biosecurity recommendations, published scientific evidence on the effectiveness of individual biosecurity measures is limited. The objective of this study was to assess the perception of Swiss experts about the effectiveness and importance of individual on-farm biosecurity measures for cattle and swine farms (31 and 30 measures, respectively). Using a modified Delphi method, 16 Swiss livestock disease specialists (8 for each species) were interviewed. The experts were asked to rank biosecurity measures that were written on cards, by allocating a score from 0 (lowest) to 5 (highest). Experts ranked biosecurity measures based on their importance related to Swiss legislation, feasibility, as well as the effort required for implementation and the benefit of each biosecurity measure. The experts also ranked biosecurity measures based on their effectiveness in preventing an infectious agent from entering and spreading on a farm, solely based on transmission characteristics of specific pathogens. The pathogens considered by cattle experts were those causing Bluetongue (BT), Bovine Viral Diarrhea (BVD), Foot and Mouth Disease (FMD) and Infectious Bovine Rhinotracheitis (IBR). Swine experts expressed their opinion on the pathogens causing African Swine Fever (ASF), Enzootic Pneumonia (EP), Porcine Reproductive and Respiratory Syndrome (PRRS), as well as FMD. For cattle farms, biosecurity measures that improve disease awareness of farmers were ranked as both most important and most effective. For swine farms, the most important and effective measures identified were those related to animal movements. Among all single measures evaluated, education of farmers was perceived by the experts to be the most important and effective for protecting both Swiss cattle and swine farms from disease. The findings of this study provide an important basis for recommendation to farmers and policy makers.

## Introduction

Within the context of livestock production, biosecurity is defined as management activities that reduce the opportunities for infectious agents to gain access to, or spread within, a production unit [1]. In Switzerland and other European countries, biosecurity is achieved through a combination of nationally legislated and voluntary on-farm measures. The significance of on-farm biosecurity has been emphasized in the European Union Animal Health Strategy 2007–2013 “Prevention is Better than Cure”. Within the first draft of the new Animal Health Law of the European Union, an attempt was made to focus on on-farm biosecurity in order to allow free trade across the borders of different European countries [2]. This strategy therefore commits farmers to maintaining high on-farm biosecurity standards.

Biosecurity practices might differ among and within countries for reasons such as differences in production types, diseases present, legislation on disease control, and available resources. The Swiss approach to maintaining a disease-free livestock population is dominated largely by governmental control measures, with the compulsory Bluetongue (BT) vaccination in 2008–2010 and the ongoing Bovine Virus Diarrhea (BVD) eradication program being notable examples [3,4]. In contrast, the implementation of on-farm biosecurity measures in Switzerland is relatively poor. This may be associated with the fact that Swiss livestock herds are still small, despite the global trend towards fewer and bigger enterprises. In 2011, the average herd size of a Swiss cattle farm was 39 (33 in 2001), and that of a Swiss swine farm 190 (105 in 2001) [5]. Larger holdings are more likely to suffer greater economic losses in the event of a disease outbreak. This may be one reason why larger enterprises apply a stricter biosecurity management than small and backyard holdings [6–8]. Despite the poor implementation of on-farm biosecurity measures, Switzerland has maintained a favorable animal disease status [9]. This favorable status might further contribute to a more relaxed biosecurity attitude of farmers [10].

There are many studies reporting biosecurity measures commonly implemented, as well as the factors that influence implementation of biosecurity measures by farmers and veterinarians [7,10–18]. Also many studies exist that describe which measures should be applied in order to keep disease risk at a minimum [19–22]. However, these studies are often only based on general knowledge about infectious diseases. Furthermore, it has been suggested that the large variety of published recommendations, might confuse and thus discourage farmers from implementing biosecurity measures [23,24]. This may explain why there is a great deal of variation in, and even absence of on-farm biosecurity practices as observed in some studies [8,23]. The limited examples of proven efficacies, combined with the lack of relevant education are potential reasons for infrequent or non-compliance to biosecurity measures [23]. All these factors may contribute to the negative attitude farmers often have towards biosecurity [8,25]. Educating farmers about biosecurity is an important factor influencing the implementation of biosecurity measures [11,26,27]. When communicating to farmers, it is essential to describe the protective effect and potential benefit of various biosecurity measures. This allows farmers to focus on biosecurity measures that are relevant to their production type, and the disease risks they face, thus optimizing time and resource expenditures. The effectiveness of biosecurity measures has been reported in an observational study that investigated disease spread relative to recommended biosecurity measures during an outbreak [28]. However, this is difficult to demonstrate in the field in the absence of a disease outbreak in a region. Controlled, experimental settings are not an optimal approach, because it is a challenge to extrapolate the results to field conditions. Metrics for quantifying the effectiveness of biosecurity measures in field settings include calculating the basic reproductive rate of an infection (R0) for different scenarios [29] or estimating the population attributable fraction (PAF) of disease for each biosecurity measure. The latter is defined as the fraction of disease in the population that could be prevented through elimination of a risk factor, i.e. the implementation of a biosecurity measure [30]. These methods are rarely used because of the challenge of correlating different measures among themselves and with external factors in field studies [31].

Expert opinions are a valuable option for gathering knowledge in a field where accurate and unbiased field data is unavailable [32]. The Delphi technique in particular, is commonly used to generate consensus amongst experts, and has also found application in veterinary epidemiology [33,34]. The Delphi technique has been criticized for generating subtle pressure to conform with group consensus, which may lead to a watered-down best opinion [35]. In addition, the method can sometimes be time consuming [35,36]. Nevertheless, if planned and implemented carefully, the Delphi technique can be very useful for capturing information upon which to base policy decisions. One of the advantages of the Delphi method is that experts can be questioned independently allowing each expert opinion to be weighted equally [37]. Furthermore, the feedback of the group consensus and re-evaluation of the experts own answer reduces the overall variance while avoiding the social and personality influences that may arise in group discussions.

The aim of this study was to assess the perception of Swiss experts about the effectiveness and importance of individual on-farm biosecurity measures for cattle and swine farms (31 and 30 measures, respectively), using a modified Delphi method. The study results will have value for developing biosecurity recommendations for farmers and informing risk based surveillance and disease control policy.

## Materials and Methods

### Expert opinion

A modified Delphi method was used in this study [37,38]. The expert opinion consisted of face-to-face interviews, followed by a report with the initial findings, and telephone calls for the discussion and revision of the results.

### Selection of experts

Our goal for selecting experts was to include a broad range of veterinary expertise from the field of animal disease control in Switzerland. Experts were employed in public veterinary services (n = 6), universities (n = 6), or animal health institutions (n = 4) in Switzerland. These were the experts most likely to be consulted in the event of an infectious disease outbreak in livestock within Switzerland. All cattle and swine experts that were contacted (eight for each species) agreed to participate in the study. Each expert was interviewed at a location of his choice and all interviews were conducted by the same interviewer, namely the first author.

### Selection of on-farm biosecurity measures

Based on a thorough review of literature, measures aimed at preventing transmission of infectious agents with varying transmission characteristics were selected and a list of on-farm biosecurity measures was created. Biosecurity measures were grouped into 11 categories on the basis of common vehicles and modes of transmission or prevention of infectious agents. A final list of 32 on-farm biosecurity measures, of which 31 were applicable to cattle farms (Table 1) and 30 to swine farms (Table 2) were selected. “Vaccination”, which fits in the category “reduction of infection pressure” was included as a separate category and was evaluated for those diseases where vaccination can be applied.

**Table 1:** Perceived importance and effectiveness of on-farm biosecurity measures for cattle farms.

Biosecurity category and measure	Perceived Importance	Perceived Effectiveness on			
		BT	BVD	IBR	FMD
*1*. *Category*: *Animal movement*					
Minimize purchase and sale of animals	3 (2.5–4)	2 (0–3)	4 (3–5)	3.5 (2–5)	3.5 (1–5)
Purchase from farms with known disease status or health certificate	4 (3.5–5)	3 (1–5)	5 (5)^a^	5 (4–5)^a^	4.25 (3–5)
Quarantine facility for sick animals and new arrivals	4.25 (3–5)	2 (0–3)	3.5 (2–5)	3.75 (2–5)	3 (2–5)
Quarantine animals after market/show	2.75 (1–4)	1 (0–3)	3 (1–5)	3.5 (2–5)	4 (2–5)
Closed herd or all-in-all-out replacement	2.5 (2–4)	1 (0–2)	2.5 (1–5)	3 (0–5)^b^	2 (0–5)^b^
**Median Score**	**4**	**2**	**4**	**4**	**3**
*2*. *Category*: *Animal contacts*					
Separation of pastures of neighboring farms	2.5 (0–4)	0 (0–1)^a^	4 (1–5)	3.25 (1–5)	2.5 (0–4)
Measures (Testing, only healthy animals on summer pasture) for common summer pastures	4.25 (4–5)^a^	2 (1–3)	5 (5)^a^	4 (3–5) ^a^	3 (0–4)
Prevention of contact with wild animals	1.5 (0–2.5)	1 (0–2)	1 (0–3)	1.5 (0–3.5)	3 (1–4)
Prevention of contact with pets	1 (0–2.5)	0 (0–1)^a^	0 (0–1)^a^	0 (0–1)^a^	0 (0–2)
**Median Score**	**2**	**1**	**2**	**3**	**2**
*3*. *Category*: *Farmers/Workers*					
Farmer/Worker has no contact with cloven-hoofed animals from other farms	2.5 (1–4)	0.5 (0–2)	3 (1–4)	3 (1–4)	4.75 (3–5)
Personal working hygiene of farmer/worker (boots, clothes, hands,…)	5 (2–5)	1 (0–2.5)	3.5 (2–5)	2.5 (2–5)	2.75 (1–5)
**Median Score**	**3**	**1**	**3**	**3**	**4**
*4*. *Category*: *Visitors*					
Access restriction for visitors	3.75 (1–4)	0 (0–1)^a^	1.5 (0–3)	3.25 (0–5)^b^	3.5 (1–5)
In-house or clean boots and clothes for non-professional visitors	4 (2–5)	0 (0–1)^a^	4 (1–5)	3.25 (1–5)	4 (2.5–5)
Personal working hygiene of professional visitors (boots, clothes, hands,. . .)	5 (3–5)	0.5 (0–1)^a^	4.5 (3–5)	3.75 (2–5)	4.5 (2.5–5)
**Median Score**	**4**	**0**	**3.5**	**3.5**	**4**
*5*. *Category*: *Vehicles*					
Vehicle access restriction	2.25 (0–4)	0 (0–1)^a^	1 (1–3)	1.5 (0–3)	4 (2–5)
Animal transport vehicle leak-proof	4 (1–5)	1 (0–1)^a^	2 (2–4)	3.75 (0–5)^b^	3.5 (2–5)
Cleaning and disinfection of the vehicle	4.75 (4–5)^a^	1 (0–2)	4 (3–5)	3.75 (2–5)	4.5 (2–5)
**Median Score**	**4**	**1**	**2.5**	**3**	**4**
*6*. *Category*: *Stable*					
Arthropod control	3 (0–4)	4.75 (3–5)	0 (0–1)^a^	0.5 (0–2)	1 (0–2)
Rodent control	2.25 (0–3)	0 (0–1)^a^	0 (0–1)^a^	0.5 (0–2)	1 (0–4)
**Median Score**	**2.75**	**2**	**0**	**0.5**	**1**
*7*. *Category*: *Feedstuff*					
Treatment of feedstuff (chemically, physically)	0.5 (0–5)^b^	0 (0–1)^a^	0 (0)^a^	0 (0–1)^a^	0.5 (0–1)^a^
Storage of feedstuff dry and protected	2.5 (0–5)^b^	0 (0–1)^a^	0 (0–1)^a^	0 (0–1)^a^	0 (0–1)^a^
**Median Score**	**1.5**	**0**	**0**	**0**	**0**
*8*. *Category*: *Disease awareness*					
Education for animal keepers (raising disease awareness)	5 (3–5)	5 (3.5–5)	4.5 (4–5)^a^	4 (3–5)	4.5 (2.5–5)
Animal health monitoring by the farmer	4.5 (3–5)	3 (2.5–5)	4 (2–5)	3.5 (2–5)	4.5 (2.5–5)
**Median Score**	**5**	**4.25**	**4**	**4**	**4.25**
*9*. *Category*: *Reduction of infection pressure*					
Limitation of number of animals	1.75 (0–5) ^b^	1.5 (0–5) ^b^	1 (0–4)	0 (0–4)	2.5 (0–4)
Good health management	4.5 (3–5)	3 (1–5)	3 (2–5)	3 (1–5)	3 (2–5)
Disposal of carcasses and manure	4 (2–5)	1 (0–2)	2.5 (0–5)^b^	1.5 (0–5)^b^	3 (1–5)
**Median Score**	**3.5**	**2**	**2**	**2**	**3**
*10*. *Category*: *Contact to the outside world*					
Closed housing	1 (0–3)	4 (3–5)	1 (0–2)	1 (0–4)	3.5 (0–5)^b^
Geographical barriers (mountains, rivers,. . .)	0 (0–4)	2.75 (0.5–5)	0.5 (0–1)	1 (0–4)	2.5 (0–5)^b^
Low animal density in the area	2 (0–3.5)	3 (0–5)^b^	2 (0–3)	0.5 (0–2)	3 (0–4)
No breeding animals, transport vehicles and equipment shared with other farms	2.5 (0–4)	1 (0–5)^b^	4 (0–5)^b^	4.5 (3–5)	4.5 (0–5)^b^
**Median Score**	**1**	**3**	**1**	**1**	**3**
*11*. *Vaccination*					
BT-Vaccination	1.5 (0–4)	5 (4.5–5)^a^			
BVD-Vaccination	0 (0)^a^		1 (0–4)		
IBR-Vaccination	0 (0)^a^			2 (1–5)	
FMD-Vaccination	0 (0)^a^				4 (3–5)

^a^Measures showing a strong agreement (maximum 1 score of difference)

^b^Measures showing a maximal disagreement (maximum 5 scores of difference)

Median and range (maximum 0–5) of values of the scores of Swiss cattle experts are shown as well as overall median for each biosecurity category.

**Table 2:** Perceived importance and effectiveness of on-farm biosecurity measures for swine farms.

Biosecurity category and measure	Perceived Importance	Perceived Effectiveness on			
		ASF	EP	FMD	PRRS
*1*. *Category*: *Animal movement*					
Minimize purchase and sale of animals	3.5 (2–5)	4.5 (3–5)	4 (3–5)	5 (3–5)	4.5 (3–5)
Purchase from farms with known disease status or health certificate	5 (2–5)	5 (3–5)	5 (4–5)^a^	5 (3–5)	5 (3–5)
Quarantine facility for sick animals and new arrivals	5 (4–5)^a^	4 (2–5)	4 (3–5)	4 (1–5)	5 (3–5)
Quarantine animals after market/show	5 (4–5)^a^	5 (4–5)^a^	5 (4–5)^a^	5 (4–5)^a^	5 (5)^a^
Closed herd or all-in-all-out replacement	4 (2–5)	5 (3–5)	4.5 (4–5)^a^	4 (3–5)	5 (4–5)^a^
**Median Score**	**5**	**5**	**4**	**4**	**5**
*2*. *Category*: *Animal contacts*					
Prevention of contact with wild animals	4 (2–5)	5 (3–5)	5 (3–5)	3 (1–5)	2.75 (0–5)^b^
Prevention of contact with pets	2.5 (0–4)	1 (0–2)	1 (0–3)	1 (0–4)	2 (0–4)
**Median Score**	**3**	**2.5**	**3**	**2**	**2**
*3*. *Category*: *Farmers/Workers*					
Farmer/Worker has no contact with cloven-hoofed animals from other farms	3.75 (2–5)	4.5 (3–5)	4 (2–5)	4.5 (3–5)	4 (2–5)
Personal working hygiene of farmer/worker (boots, clothes, hands,…)	4 (2–5)	3 (2–5)	3 (2–4)	3.5 (1–5)	4 (3–5)
**Median Score**	**4**	**4**	**3**	**4**	**4**
*4*. *Category*: *Visitors*					
Access restriction for visitors	3.25 (2–5)	3 (1–5)	2 (0–3)	4 (2–5)	3.5 (1–5)
In-house or clean boots and clothes for non-professional visitors	3.25 (2–5)	3.5 (1–5)	2 (1–5)	3.5 (2–5)	3 (2–5)
Personal working hygiene of professional visitors (boots, clothes, hands,. . .)	4 (4–5)^a^	4 (3–5)	3 (3–5)	4 (2–5)	4.5 (3–5)
**Median Score**	**4**	**4**	**3**	**4**	**4**
*5*. *Category*: *Vehicles*					
Vehicle access restriction	2 (0–5)^b^	4 (1–5)	2 (1–5)	4.5 (3–5)	4 (1–5)
Animal transport vehicle leak-proof	4 (3–5)	4 (2–5)	2.5 (0–5)^b^	5 (3–5)	4 (2–5)
Cleaning and disinfection of the vehicle	4.75 (3–5)	5 (3–5)	4 (3–5)	5 (3–5)	5 (3–5)
**Median Score**	**4**	**4**	**3**	**5**	**4**
*6*. *Category*: *Stable*					
Cleaning and disinfection of the compartments following animal replacement	4 (2–5)	2 (1–3)	2.75 (2–4)	1.5 (1–4)	2.5 (1–4)
Arthropod control	3 (2–4)	3 (1–5)	1.5 (0–4)	3 (0–4)	3 (1–4)
Rodent control	4 (2–5)	2.75 (0–4)	1.5 (0–4)	2 (1–4)	2 (0–4)
**Median Score**	**4**	**2.75**	**2**	**2**	**3**
*7*. *Category*: *Feedstuff*					
Treatment of feedstuff (chemically, physically)	1 (0–3)	1 (0–5)^b^	0 (0–1)^a^	1.5 (0–5)^b^	1 (0–2)
Storage of feedstuff dry and protected	1.5 (1–5)	1 (0–2.5)	0 (0–1)^a^	1 (0–3)	1 (0–1)^a^
**Median Score**	**1**	**1**	**0**	**1**	**1**
*8*. *Category*: *Disease awareness*					
Education for animal keepers (raising disease awareness)	4 (2–5)	4 (1.5–5)	3.5 (2–5)	4 (3–5)	4 (3–5)
Animal health monitoring by the farmer	4 (1.5–5)	4.5 (1–5)	3 (1.5–5)	3.5 (2–5)	4 (2–5)
**Median Score**	**4**	**4**	**3**	**4**	**4**
*9*. *Category*: *Reduction of infection pressure*					
Limitation of number of animals	2 (0–5)^b^	1.5 (0–4)	4 (0–5)^b^	2 (0–3)	3 (0–4)
Good health management	3.75 (2–5)	2 (1–4)	3 (0–5)^b^	2.5 (1–4)	4 (2–5)
Disposal of carcasses and manure	4 (2.5–4)	3.5 (2–5)	1.5 (0–3)	4 (1–5)	2.75 (1–5)
**Median Score**	**3.5**	**2.25**	**2**	**3**	**3**
*10*. *Category*: *Contact to the outside world*					
Closed housing	3 (0–5)^b^	4 (3–5)	3.5 (2–5)	4 (3–5)	4.5 (2–5)
Geographical barriers (mountains, rivers,. . .)	2 (0–5)^b^	2.5 (0–4)	4 (2–5)	3 (0–5)^b^	4.25 (0–5)^b^
Low animal density in the area	3.75 (2–5)	3 (0–5)^b^	4 (3–5)	4.5 (2–5)	4.5 (2–5)
No breeding animals, transport vehicles and equipment shared with other farms	4.75 (4–5)^a^	5 (4–5)^a^	5 (4–5)^a^	5 (4–5)^a^	5 (5)^a^
**Median Score**	**4**	**4**	**4**	**4**	**5**
*11*. *Vaccination*					
EP-Vaccination	0 (0–1)^a^		2 (0–4)		
FMD-Vaccination	0 (0–1)^a^			4 (4)^a^	
PRRS-Vaccination	0 (0–1)^a^				2.5 (1–4)

^a^Measures showing a strong agreement (maximum 1 score of difference)

^b^Measures showing a maximal disagreement (maximum 5 scores of difference)

Median and range (maximum 0–5) of values of the scores of Swiss swine experts are shown, as well as overall median for each biosecurity category.

### Selection of diseases

As some routes of transmission are more relevant for one disease than for another, we focused the evaluation of the perceived effectiveness on specific diseases. This would allow the prioritization of biosecurity measures for specific or related diseases. Furthermore, this would also facilitate a more precise evaluation by the experts. Individual diseases were selected to be representative of: diseases with vector-borne transmission (Bluetongue, BT), (re-) emerging diseases (African Swine Fever, ASF), those having a high economic impact (Foot and Mouth Disease, FMD), and diseases that are particularly relevant for Switzerland. The latter, are either officially eradicated, and only appear sporadically (Enzootic Pneumonia, EP; Porcine Reproductive and Respiratory Syndrome, PRRS; Infectious Bovine Rhinotracheitis, IBR), or are subject to an ongoing eradication program (Bovine Viral Diarrhea, BVD). Cattle experts were asked to rank the effectiveness of biosecurity measures for dealing with the pathogens causing BT, BVD, IBR and FMD, while swine experts were asked to rank those related to ASF, EP, PRRS and FMD.

### Interviews and reports

The set-up was designed based on the experience of four pilot interviews to minimize question ambiguity and generally refine the opinion process. The face-to-face interviews of the Swiss experts were conducted from February to April 2012. According to Swiss legislation, no ethical approval was required for this study since no sensitive data were collected. The research objectives of the study were communicated to all participants and their agreement to participate was obtained through a written consent. All experts were assured anonymity. Interviews were audiotaped for documentation. During these interviews, experts were given the task of ranking biosecurity measures based on their perceived effectiveness and importance of each measure in preventing infectious agents from entering and spreading within, a farm. The diseases to be evaluated for the perceived effectiveness were assigned to the experts in random order.

Each biosecurity measure was written on a card. For some measures, a brief explanation of the measure was included on the back of the card, to ensure that all experts used the same definition for each measure. As an example, for the biosecurity measure “animal health monitoring by the farmer”, the explanation “farmer observes and knows his/her animals; he/she keeps records of disease occurrence and treatments” was provided. During the interview, the cards were shuffled and handed over to the experts for ranking. Experts were first asked to sort the biosecurity measure cards along an arrow, from “no importance” to “of utmost importance”. In a second step, the experts were asked to allocate a score from 0–5 to each biosecurity measure card. This approach was intended to assist experts by helping them focus on ranking the measures first, and then assign a semi-quantitative value to the ranked measures. A 6-point scale was used to necessitate the experts to lean towards one of the given scale extremities and not to remain in neutral position. For the assessment of the perceived effectiveness, it was left up to the expert to choose whether to use the scale from 0–5 immediately, or in a second step.

The first task given to the experts was to rank the cards based on their perceived importance of each measure in preventing an infectious agent from entering and spreading within a farm. For this, the experts were asked to consider the feasibility, effort required, and benefit of each measure, as well as Swiss legislation. With the definition of “importance”, we intended to collect the opinion of the experts on the biosecurity measures that should be promoted within Switzerland. The second task was to rank the perceived effectiveness of the measures in preventing specific infectious agents from entering and spreading within a farm. Experts were asked to base this ranking solely on the transmission characteristics of the particular agents, irrespective of the feasibility of the measure and the prevalence of the disease.

The experts were allowed to write down and rank additional biosecurity measures if they thought the list was incomplete or not sufficiently precise. They were also allowed to ask questions at any time during the interview. In order to get an impression of how experts perceive their knowledge, they were asked to assess their knowledge about each disease, using a score from 1 (poorest) to 6 (best). The results of the self-evaluation were not considered in the analysis.

Following the completion of all 16 interviews, a report was sent to each expert per mail. Each report contained five bar charts (one for the perceived importance, and one for the perceived effectiveness towards each of the four diseases), showing the individual experts’ scores and the median and range of the scores from all experts for the same species. In a second round of the expert opinion, each expert was given the opportunity to revise his scores during a pre-arranged phone call. Changes were documented, and the revised scores were used in the final analysis.

### Statistical analysis

Expert scores were reported as medians, quartiles and ranges. Perceived effectiveness of each biosecurity measure was first described for each individual disease. In addition, the median of the perceived effectiveness scores of all 4 diseases was calculated for each biosecurity measure and expert to describe its overall perceived effectiveness. Spearman’s rank correlation coefficient (r_s_) was used to assess the association between scores for perceived importance and effectiveness of different biosecurity measures.

To assess agreement among the different experts, the absolute value of the difference between scores was calculated for each pair of experts and each biosecurity measure. This resulted in 28 individual comparisons per biosecurity measure, up to 868 comparisons per disease for the perceived effectiveness of biosecurity measures and up to 952 comparisons for the perceived importance of biosecurity measures. Percent of comparisons with total agreement between 2 experts (difference of 0), and deviation by different amounts between 2 experts (difference from 0.5 to 5) were used to describe the raw agreement among experts. The proportion of the total variance in scores that could be attributed to individual experts was estimated with Intraclass Correlation Coefficients (ICC) from nonparametric repeated measures ANOVA models. In these models, the outcomes were the scores on perceived importance and effectiveness for the different diseases and animal species. For the calculation of ICC expert, the individual biosecurity measures were entered as a subject variable, and the experts were entered as a random effect. The ICC for contribution of experts to the total variance of scores was calculated from the model output [39]:

$$\text{ICC}=\frac{\text{Mean Square}\;(\text{Expert})-\text{Mean Square}\;(\text{Residual})}{\text{Mean Square}\;(\text{Expert})+(\text{k}-1)\;\text{Mean Square}\;(\text{Residual})}$$

k-1 represents the degrees of freedom for the model. The Wilcoxon-Mann-Whitney-test was applied to investigate differences in the median scores for the perceived importance of biosecurity measures between cattle and swine. All statistical analyses were performed with the software NCSS 8 [40]. Results were recorded in a Microsoft Access 2010 file (Microsoft Corp, Redmond, Washington USA).

## Results

The individual interviews lasted from 25 to 98 min (overall mean 53 min, 47 min for cattle experts, and 59 min for swine experts). Some experts commented that the modified Delphi method used in the study was convenient for capturing their opinions. Most experts (14/16) agreed that the list of biosecurity measures was exhaustive. Two swine experts provided the following additional biosecurity measures: “prevention of contact with birds”, “no attendance at markets/shows or no return from there”, “chronology of animal transports”, “big distance between farm and road”, “restrictions to workers having been abroad” and “buying semen only from males with health certificate”. Since these measures were evaluated only by the experts who suggested them, they were not included in the second round of opinion or in the final study results. In the second round of the opinion process, all the experts reassessed their scores. The reassessment however did not result in any changes in the median values.

**Fig 1:**
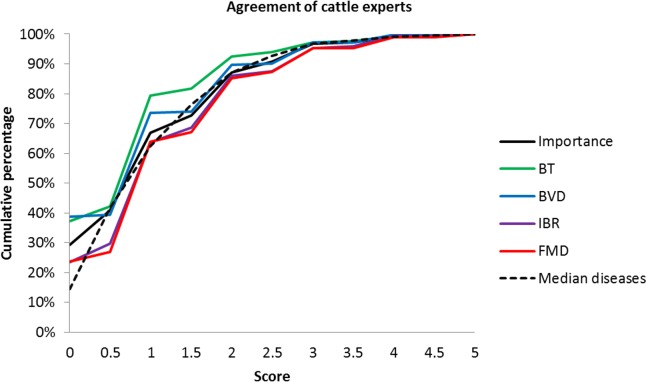
Agreement of cattle experts’ opinions of 31 biosecurity measures. Cumulative percentage of pairs of experts with a difference in scores between 0 and 5 are shown for the evaluation of the perceived importance of biosecurity measures, the perceived effectiveness of biosecurity measures against BT, BVD, IBR and FMD, as well as for the median value of the four diseases.

**Fig 2:**
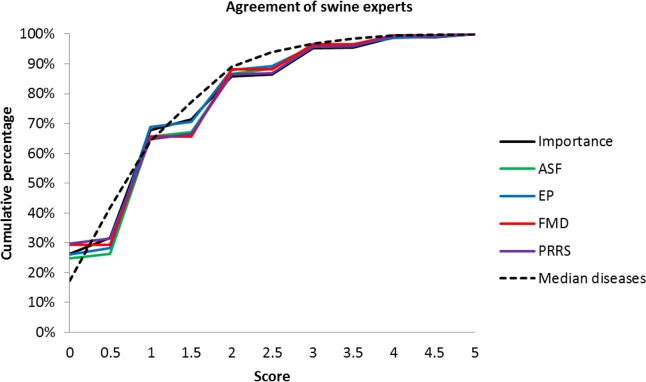
Agreement of swine experts’ opinions on 30 biosecurity measures. Cumulative percentage of pairs of experts with a difference in scores between 0 and 5 are shown for the evaluation of the perceived importance of biosecurity measures, the perceived effectiveness of biosecurity measures against ASF, EP, FMD and PRRS, as well as for the median value of the four diseases.

The most important and effective biosecurity measures for cattle farms where considered those related to “disease awareness”. For swine farms, biosecurity measures related to “animal movements” received the highest scores for their perceived importance and effectiveness. For both species, the least important and effective measures as perceived by the experts where those in the category “feedstuff”. Furthermore, vaccination was rated as being of low importance for both species. The overall assessment of the perceived effectiveness and importance of biosecurity measures is presented in Tables 1 and 2. The degree of agreement among experts ranged from complete agreement to a strong disagreement (scores ranging from 0–5). For cattle farms, experts were in almost complete agreement on the perceived effectiveness of the measure “prevention of contact with pets”, and on measures related to “feedstuff”. On the other hand, their opinions on the importance of measures related to feedstuff were divided. Cattle experts disagreed strongly on the biosecurity measures: “no breeding animals, transport vehicles and equipment shared with other farms” and “limitation of number of animals”. Swine experts agreed in their assessment of “quarantine animals after market/show” and of “no breeding animals, transport vehicles and equipment shared with other farms”. On the other hand, “geographical barriers”, “limitation of number of animals” and “vehicle access restriction”, were assessed dissimilarly. The overall differences in the assessment are shown in Figs 1 and 2. Differences of half points were not common since the instruction for the opinion was to provide scores from zero to five, and only some experts gave half points. Of all individual assessments per species, 69% for cattle and 67% for pigs differed by a maximum of one point and, 88% for cattle and 87% for pigs differed by a maximum of two points. The agreement in the assessments was comparable for the individual diseases, which indicates that one disease was not discussed more controversially than another.

The comparison of the same biosecurity measures for cattle and swine farms revealed a statistically significant difference between the perceived importance of the following biosecurity measures: “quarantine animals after market/show”, “prevention of contact with wild animals”, rodent control”, “low animal density in the area”, “geographical barriers” and “no breeding animals, transport vehicles and equipment shared with other farms” (all Wilcoxon-Mann-Whitney-test *P*-value < 0.05).

Cattle experts assessed their personal knowledge about BT (median 5.5; range 5–6) as being the best, followed by BVD (5.25; 5–6), IBR (5; 4–6) and FMD (5; 3–6). Swine experts ranked their knowledge on each disease as follows: EP (5.5; 2–6), FMD (5; 4–5.5), PRRS (5; 4–5.5) and ASF (4.5; 3–5).

For cattle biosecurity measures, there was a strong correlation between the assessment of the perceived effectiveness of measures for BVD and IBR (r_s_ = 0.8). There was a moderate correlation between the perceived importance and the perceived effectiveness of measures for BVD (r_s_ = 0.6), IBR (r_s_ = 0.6) and the overall disease median (r_s_ = 0.5). The assessment of the perceived effectiveness of the measures for FMD was also moderately correlated to those for IBR (r_s_ = 0.6) and BVD (r_s_ = 0.6).

For swine biosecurity measures, there was a strong correlation between the perceived effectiveness of measures for FMD and ASF (r_s_ = 0.8). There was a moderate correlation between the perceived importance and the perceived effectiveness of measures for ASF (r_s_ = 0.6) and for PRRS (r_s_ = 0.6).

The assessment of reliability revealed a weak dependency of the opinion results on the individual expert (ICC expert: 0.05–0.36), and a stronger dependency on the individual measures (ICC measure: 0.46–0.73) (Table 3).

**Table 3:** Intraclass Correlation Coefficients (ICC), showing the influence of the experts and biosecurity measures on the assessment of the perceived importance and effectiveness.

	ICC expert	ICC measure
**Cattle**		
Importance	0.099	0.508
BT	0.286	0.729
BVD	0.088	0.708
IBR	0.278	0.631
FMD	0.360	0.553
Median diseases	0.280	0.628
**Swine**		
Importance	0.049	0.464
ASF	0.169	0.510
EP	0.160	0.550
FMD	0.288	0.488
PRRS	0.122	0.484
Median diseases	0.124	0.516

## Discussion

This study was conducted to assess the perceptions of veterinary experts on the effectiveness and importance of individual on-farm biosecurity measures, using a modified Delphi method. For cattle farms, biosecurity measures in the category “disease awareness” of farmers were rated as being the most important (score: 5) and among the most effective (scores: 4–4.5) measures. Farmers are often the first to recognize and report disease outbreaks, and education of farmers is a fundamental tool in disease eradication [41]. For highly contagious diseases in particular, early detection and notification by farmers may have an enormous impact on disease mitigation. Other measures perceived as important (and effective for individual diseases) for cattle farms included “vehicle cleaning and disinfection”, measures on “personal working hygiene” for both, farmers and visitors, “quarantine of sick and new animals” and ensuring that only healthy animals are brought to common pastures. The latter is especially relevant for Switzerland, since alpine pasturing has been implicated in the spread of diseases such as BVD [42,43], which also explains the high perceived effectiveness score this measure has received for this particular disease.

Vaccination was rated as being of no or low importance for all cattle diseases. This was to be expected since Switzerland generally implements a non-vaccination policy. However, for BT virus this was surprising as vaccination led to a notable reduction of BT outbreaks from 2008 on, following the introduction of the disease in 2007 [3]. Since at the time of the expert opinion BT was already eradicated in Switzerland, one might assume that the experts downgraded the importance of vaccination against this disease. Nevertheless, vaccination against BT virus was still rated as effective, which might reflect that the experts are aware of the protective potential of the vaccine, but do not wish to vaccinate at all. The biosecurity category “contact to the outside world” was also rated as being of low importance for cattle farms. This is not surprising since free ranging is a common practice for cattle farming in Switzerland. Nevertheless, maximum disagreement was observed on the perception of the effectiveness of individual measures within this category, especially for FMD, which might have resulted from differences in expert knowledge.

For swine farms, measures relating to animal movements were perceived as being the most important and effective. In a country like Switzerland, where movements and mixing of pigs are generally very intensive, this was to be expected. Indeed, movements of domestic and wild animals play a central role in the spread of diseases [44]. As for cattle diseases, vaccination of pigs was rated as not being important. Prevention of contact with wild animals was rated as very effective for ASF and EP, which can be explained by the role wild boars might play in the transmission of these two diseases; however, the role of wild boars in the persistence of these pathogens in pigs is being questioned [45,46].

The perceived importance of some biosecurity measures differed significantly between cattle and swine experts; in all of these cases, higher scores were given by the swine experts. This, in turn, might reflect the stronger focus on biosecurity for swine farms. As an example, for the measure “quarantine animals after market/show”, this is possibly related to the fact that, at least in Switzerland, it is considered unacceptably risky to bring back to the farm pigs that have been to exhibitions; in the rare cases that this is done, strict quarantine measures have to be applied. The measure “prevention of contact with wild animals” is again more relevant to swine biosecurity because of the risks imposed by the increasing number of wild boars in Switzerland [47]. The same could indirectly be true for “geographical barriers”, which can restrict movements of wild boars. “Rodent control” might reflect the risk for transmission by rodents of *Salmonella* species, *Yersinia* species and other important zoonotic pathogens [48]. The measure “low animal density in the area” could be linked to the potential for disease outbreaks in areas of high livestock density, as was the case with CSF in The Netherlands [49]. The recent outbreak of PRRS caused by the import of boar semen from Germany into Switzerland might account for the high scoring of the perceived importance of the measure “no breeding animals, transport vehicles and equipment shared with other farms” by swine experts [50].

Despite some, mostly disease specific differences, the overall tendency of the experts was to rate the majority of the measures as being rather effective. Of all diseases, FMD in cattle, as well as PRRS and FMD in pigs, have received the highest values on the effectiveness of individual measures, whereas biosecurity appears according to the experts not to be very effective for vector-borne diseases such as BT. The strong correlation between the assessment of the perceived effectiveness for BVD and IBR in cattle, as well as for ASF and FMD in pigs (both with r_s_ = 0.8) might reflect the similarities in the epidemiology of these diseases. The estimation of the effectiveness of biosecurity measures has been reported to be negatively correlated to the potential for aerosol transmission [18]. This was not observed in the present study, in which measures were perceived as being the most effective on FMD virus, a pathogen that can readily be transmitted through the air over great distances [51]. Since FMD is a highly contagious and a much-feared disease, countermeasures are very important, but some might not be very effective in preventing aerosol spread. A possible explanation for the high scoring of the perceived effectiveness of biosecurity measures by the Swiss experts is that, people who have never experienced a particular disease and do not feel endangered thereof, tend to assess the effectiveness of biosecurity measures higher than people who have experienced an outbreak [18]. Switzerland is officially declared free from five (BT, FMD, ASF, PRRS, IBR) of the seven diseases investigated in this study.

Expert opinions are an established method for gathering knowledge in the absence of data. In our study, it was not surprising to observe that, recently emerged or endemic diseases in Switzerland such as BT and EP were perceived by the experts as best known, whereas exotic diseases such as ASF were perceived as the least well known. In other studies, the results of the self-evaluation have been used to weight the assessment scores [52]. We decided against it, in the belief that the subjectivity of a self-evaluation outweighs its information content. In addition, self-evaluation might not be a reliable predictor of expert performance [32].

We decided not to include any weighting in this study however, for several biosecurity scoring systems assigning weight to individual measures was a key point [53–56]. For most biosecurity measures, deciding on a logical weighting principle is hampered by the lack of data, whereas equal weighting poses the risk of under- or overestimating the contribution of certain biosecurity measures in reducing disease transmission.

A greater number of experts would have increased the study power, however this was difficult to achieve in a small country like Switzerland, with a limited number of animal health experts. In order to capture biosecurity knowledge in Switzerland, we included veterinary experts covering a wide range of expertise within the interdisciplinary field of livestock production [32]. Broadly defined expert groups have been reported to increase the accuracy of expert advice [57] however it may also have contributed to the wide range of answers, as it was observed in this study. Furthermore, because of the limited sample size, the estimation of the perceived importance and effectiveness of the measures is not exact and the ranking of biosecurity measures is not absolute.

We calculated ICC, to investigate the influence of each expert on the variance in scores. With the ICC values for the measures being consistently larger, it can be concluded that the variance in scores was influenced more by the question itself (importance/effectiveness of a particular biosecurity measure), rather than by the personal opinion of the expert.

Inconsistency in the definition and application of the term biosecurity may also have contributed to the rather wide range of expert scores for certain measures. For some experts, the inclusion of both prevention of introduction and spread in a single definition of biosecurity, may have caused some confusion and therefore inconsistency in their answers. Some biosecurity measures were probably ranked by the experts only with regards to prevention of introduction, such as for “access restriction for visitors” or, with a stronger focus on the prevention of spread, such as for “quarantine animals after market/show”. This may have been avoided by differentiating between internal biosecurity (the prevention of spread within a herd; sometimes referred to as biocontainment) [58] and external biosecurity (the prevention of introduction of pathogens into a herd) [59]. Although both, internal and external biosecurity measures were included in our study, the distinction between the two concepts was not addressed explicitly. It was our opinion that leaving these definitions out would make the opinion process shorter, less complex and more attractive to experts and that this would outweigh potential imprecision in the study results. For the same reason, the number of measures was kept at a minimum by consolidating related measures into single measures. For example, the measure: “minimize purchase and sale of animals”, is a consolidation of two measures, one referring to “purchase” and another to “sale”. For some measures, this may have caused some confusion and introduced variability. For example, the measure “disposal of carcasses and manure” left some experts (3/16) unsatisfied as they saw a greater risk associated with carcasses than with manure. Asking experts to evaluate these two measures as one, may have resulted in the perceived importance or effectiveness of carcass disposal being underestimated, and, that of manure disposal being overestimated.

Most experts (14/16) agreed that the list of biosecurity measures was exhaustive. Additional measures were proposed by only two swine experts. Measures concerning artificial insemination were not listed, with the idea that the risk of transmitting disease when using a bull or boar for mating is even higher than with artificial insemination. Bringing new genetics into a herd always carries the risk of pathogen introduction and for this reason, reproduction is included in the categories of “animal movement”, “animal contacts” and “contact to the outside world”. The measure “quarantine animals after market/show” was especially difficult to assess for swine experts (3/8 skipped this measure) since it is uncommon in Switzerland to bring pigs back to the holding after attending markets/shows, and for biosecurity reasons it would be preferable not to attend markets/shows at all.

Another challenge arose from the lack of a common agreement about the definition of individual biosecurity measures. “Geographical barriers” is not commonly considered to be a biosecurity measure, but geographical barriers can contribute to preventing infectious agents from spreading. Similarly, “minimize purchase and sale of animals” is not always classified as a biosecurity measure; however, it is known to be of major importance for disease introduction. Quarantine is defined as the isolation of animals that are either infected or suspected of being so, or of non-infected animals that are at risk [1]. Recommendations for implementation of quarantine as a biosecurity measure vary in duration and degree of separation, making a general assessment of quarantine difficult. Some experts (2/16) in this study suggested that quarantine should be extended to include periparturient animals in addition to sick and newly introduced animals. Harmonized definitions of (on-farm) biosecurity measures would facilitate future research and the development of standardized, evidence-based recommendations.

Differentiating between perceived importance and effectiveness of measures was essential for gaining a clear understanding of the biosecurity measures that should be promoted within Switzerland. The term “importance” was introduced in order to include aspects that are country specific. For example, vaccination against FMD is effective for preventing or controlling an outbreak, but it is not considered an important measure in Switzerland, as current Swiss legislation prohibits its application. In an attempt to prevent confusion, both the definitions of importance and effectiveness, and the expert opinion process were explained to experts and provided in written form. Furthermore, experts were allowed to ask questions, and were given as much time as needed to complete their opinion. Nonetheless, the distinction between “importance” and “effectiveness” proved to be challenging to some experts, and may have contributed to the variability in some of the answers.

## Conclusion

This study provided some valuable information on the perceptions of veterinary experts on the importance and effectiveness of biosecurity measures for cattle and swine farms in Switzerland. Farmers’ disease awareness and animal movements were identified as core components for safeguarding cattle and swine farms from infectious disease. While the perceived importance of biosecurity measures is specific for Switzerland, the perceived effectiveness of these measures could also be useful to other countries since it relates to the nature of the disease and is thus not country specific. Moreover, this study has also identified some important points that need to be considered when planning an expert opinion on this field. One of the greatest challenges for the experts was the distinction between the terms “importance” and “effectiveness” of biosecurity measures. Furthermore, the study has highlighted the need for more precise and commonly accepted definitions of biosecurity measures. The partially broad range of the expert opinions also raises the demand for further research on the effectiveness of biosecurity measures. This would facilitate communication to farmers and policy makers on the value of on-farm biosecurity.
